# Characterization of a New Intracellular Alginate Lyase with Metal Ions-Tolerant and pH-Stable Properties

**DOI:** 10.3390/md18080416

**Published:** 2020-08-09

**Authors:** Yan Ma, Jie Li, Xin-Yue Zhang, Hao-Dong Ni, Feng-Biao Wang, Hai-Ying Wang, Zhi-Peng Wang

**Affiliations:** 1 Marine Science and Engineering College, Qingdao Agricultural University, Qingdao 266109, China; mayan3945@163.com (Y.M.); 17852840297@163.com (X.-Y.Z.); nhd17664081021@163.com (H.-D.N.); wfb19980903@163.com (F.-B.W.); 2 Yellow Sea Fisheries Research Institute, Chinese Academy of Fishery Sciences, Qingdao 266109, China; lijie@ysfri.ac.cn; 3 Key Laboratory of Maricultural Organism Disease Control, Ministry of Agriculture and Rural Affairs, Qingdao 266071, China; 4 Key Laboratory of Sustainable Development of Polar Fishery, Ministry of Agriculture and Rural Affairs, Qingdao 266071, China

**Keywords:** Alginate lyase, Intracellular, pH-stable, Metal ion-resisted, *Yarrowia lipolytica*

## Abstract

Alginate lyases play an important role in alginate oligosaccharides (AOS) preparation and brown seaweed processing. Many extracellular alginate lyases have been characterized to develop efficient degradation tools needed for industrial applications. However, few studies focusing on intracellular alginate lyases have been conducted. In this work, a novel intracellular alkaline alginate lyase Alyw202 from *Vibrio* sp. W2 was cloned, expressed and characterized. Secretory expression was performed in a food-grade host, *Yarrowia lipolytica*. Recombinant Alyw202 with a molecular weight of approximately 38.3 kDa exhibited the highest activity at 45 °C and more than 60% of the activity in a broad pH range of 3.0 to 10.0. Furthermore, Alyw202 showed remarkable metal ion-tolerance, NaCl independence and the capacity of degrading alginate into oligosaccharides of DP2-DP4. Due to the unique pH-stable and high salt-tolerant properties, Alyw202 has potential applications in the food and pharmaceutical industries.

## 1. Introduction

Alginate is a natural bioactive polysaccharide derived from the cell walls of widespread brown algae and several bacteria, which is composed of β-D-mannuronic acid (M) and α-L-guluronic acid (G) as monomeric units. The monomers of three kinds of different blocks are presented in the poly-β-D-mannuronic acid (polyM), poly-α-L-guluronic acid (polyG) and random heteropolymer (polyMG) [1]. The content ratio and arrangement of these monomers have a significant influence on biological properties of alginate, such as the gelatinization of fibers and the strengthening of cell walls [2]. Due to its favorable properties of biocompatibility and gelation ability, alginate polymers and its derivatives are widely used in food, pharmaceutical and cosmetic industries, especially in the field of biomedical engineering recently [3].

Alginate lyases can degrade alginate into alginate oligosaccharides (AOS) and monosaccharides by cleaving the glycosidic bond via the β-elimination mechanism [4]. AOS are the smaller bioactive units consisting of 3 to 25 monomers, which have versatile biological properties, including neuroprotective, antioxidant, anti-proliferative and antitumor capacities, as well as promoting plant growth [5,6,7,8]. To date, most functional alginate lyases are identified from alga-associated bacteria, such as *Pseudomonas aeruginosa*, *Zobellia galactanivorans* and *Flavobacterium* sp. strain UMI-01 [9,10,11], and among them some genes have been successfully cloned and sequenced. Inoue et al. first reported an alginate lyase from brown alga, which is the leading alginate producer in nature [2]. Based on their different ways of action, all these alginate lyases can be grouped into exolytic and endolytic alginate lyases. The exolytic ones can excise glyosidic bonds from the end of alginates to release monomers, while the endolytic ones cleave glyosidic bonds inside alginate randomly to form AOS [12]. Given the high efficiency and mild reaction condition, alginate lyases, especially endolytic enzymes, have recently attracted public attention. Nevertheless, most studies of alginate lyases focused on pursuing high catalytic activity, new types and analyzing the structures, limiting their utility in AOS production. Therefore, from the aspect of practical application, stable and high-efficiency alginate lyases should be identified for both research and commercial purposes.

So far, many extracellular alginate lyases secreting from bacteria have been well characterized for potential industrial applications [13,14,15,16,17,18]. However, few studies of intracellular alginate lyases have been conducted, which are also potential candidates for commercial use of alginate lyases as important as the extracellular ones. As reported previously, we have isolated a cold-adapted alginate lyase-producing strain *Vibrio* sp. W2, and the recombinant extracellular alginate lyase was also well characterized [13]. In this work, a new intracellular alginate lyase with pH-stable and high salt-tolerant properties has been identified and characterized from *Vibrio* sp. W2 strain. Its excellent catalytic performance suggests that Alyw202 can be an efficient tool for commercial utilization and industrial production.

## 2. Results and Discussion

### 2.1. Purification of the Intracellular Alginate Lyase in Vibrio sp. W2

**Figure 1:**
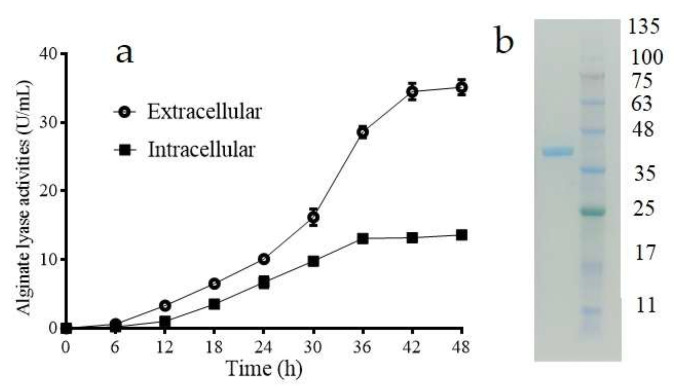
(**a**) Extracellular and intracellular enzyme activity curves of strain W2; (**b**) SDS-PAGE analysis of purified Alyw202. Lane M, prestained marker proteins; Lane 1, purified Alyw202.

As mentioned in our previous study, *Vibrio* sp. W2 was screened from the viscera of abalone using alginate as the sole carbon source at 25 °C [13]. During the 48 h fermentation in ASC liquid medium at 25 °C, alginate lyase activity was observed in culture supernatants, while no alginate-degrading activity was detcted in the collected intact cells. To check the existence of intracellular alginate lyase, the cells were broken by ultrasonic degradation in phosphate buffer solution (50 mM, pH 7.0) as much as supernatants in volume. After centrifugation, the intracellular alginate lyase was present in the supernatant. As is shown in Figure 1a, the intracellular alginate lyase activity achieved 13.6 U/mL at flask level, while the supernatant contained 35.12 U/mL of the extracellular alginate lyase activity after 48 h cultivation. At 48 h, the specific activities of extracellular alginate lyase and intracellular alginate lyase were 78.7 and 126.4 U/mg. Thus, the intracellular alginate lyase was confirmed, and concerning the endogenous synthesis, the performance of this intracellular alginate lyase was relatively satisfactory.

As shown in Table 1, the intracellular alginate lyase was purified by using DEAE-Sepharose fast flow and Sephadex G-75 chromatography. As shown in Figure 1b, the purified protein exhibited a single main band on SDS-PAGE that was consistent with a molecular weight of 38.3 kDa. The molecular weight of intracellular Alyw202 is similar to molecular weights of AlyV5 (37 kDa) from *Vibrio* sp. QY105 [19] and Aly1281 (40.65 kDa) classified as PL family 7 from *Pseudoalteromonas carrageenovora* ASY5 [20]. This intracellular alginate lyase was named Alyw202 and used in the following investigation. The specific activity of purified Alyw202 toward sodium alginate was 1926.4 U/mg, while those toward polyG blocks and polyM blocks were 2132.2 and 1074.6 U/mg, respectively. These specific activities were much higher than those of Alyw201 [13]. The results indicated that Alyw202 may be a more efficient tool for alginate degradation.

### 2.2. Bioinformatics Analysis of the Alginate Lyase Alyw202

To investigate further, it is necessary to clone the gene of Alyw202. The genome DNA of this strain was sequenced as mentioned earlier [13]. Sequence analysis revealed the presence of a single putative gene *ALYW202* (MT424751) encoding an alginate lyase without the signal peptide, consistent with the intracellular-enzyme characteristic. The open reading frame (ORF) consists of 1053 bp and encodes a protein of 350 amino acids. Further bioinformatics analysis concluded that the theoretical isoelectric point (pI) was 5.10, and molecular weight (Mw) was 38.3 kDa, consistent with the molecular weights of the Alyw202 detected by SDS-PAGE.

**Figure 2:**
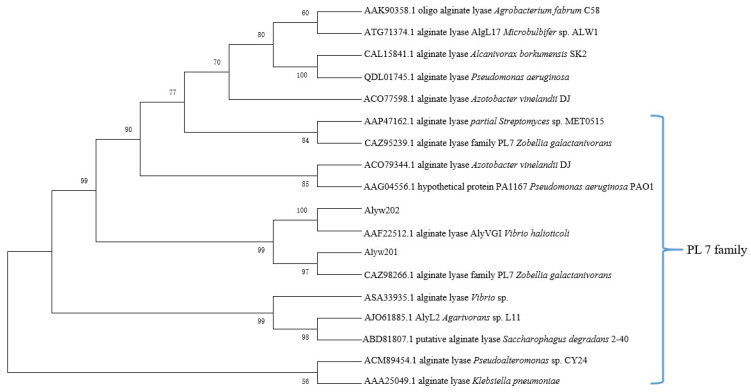
Neighbor-joining phylogenetic tree generated based on the amino acid sequences of Alyw202 and other reported alginate lyases.

**Figure 3:**
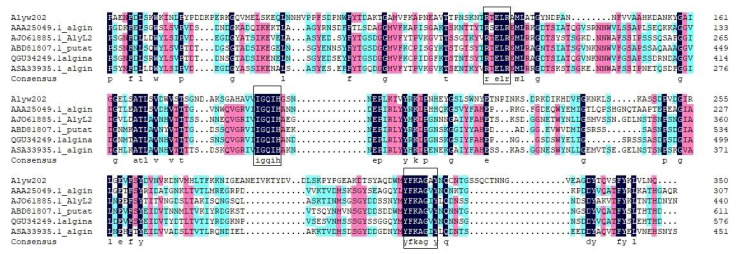
Multiple sequence alignments of Alyw202 and other five well-characterized alginate lyases from the PL7 family by using DNAMAN 6.0. The conserved domains are marked in the black box.

By blasting on NCBI, Alyw202 has one conserved domain belonging to the polysaccharide lyase (PL) family 7 and alginate lyase superfamily 2. To further determine the attribution of Alyw202, a phylogenetic tree was constructed based on the amino acid sequence of Alyw202 and other 17 reported alginate lyases from diverse PL families. According to the homology of amino acid sequences from different alginate lyases species shown in Figure 2, Alyw202 clearly belongs to the PL7 family, which forms an all beta fold and is most homologous to alginate lyase of *Vibrio halioticoli* (AAF22512.1) [21] and of *Zobellia galactanivorans* (CAZ95239.1) [10], as well as the extracellular Alyw201 of strain W2 [13]. Additionally, further sequence alignment was performed between Alyw202 and five other well-characterized alginate lyases of the PL7 family. As shown in Figure 3, these five alginate lyases respectively come from *Klebsiella pneumoniae* (Accession: AAA25049.1), *Agarivorans* sp. L11 (Accession: AJO61885.1), *Saccharophagus degradans* 2-40 (Accession: ABD81807.1), *Marinimicrobium* sp. (Accession: QGU34249.1) and *Vibrio* sp. (Accession: ASA33935.1) [22,23,24,25]. Alyw202, like the other typical alginate lyases of the PL7 family, contains the conserved regions such as “QIH” and “YFKAG” (Figure 3). These conserved regions as recognition sequence target on the specific blocks of alginate. Researchers considered that alginate lyases containing the “QIH” region prefer polyG blocks as substrate, which was already confirmed by alginate lyases from *Corynebacterium* sp. strain ALY-1 and *Sphingomonas* sp. A1 [26,27]. Both Alyw201 and Alyw202 exhibit higher activities toward polyG block [13]. This may be attributed to the common “QIH” block in the amino acid sequences of the two alginate lyases.

### 2.3. Expression of Alyw202

For better characterization, the ALYW202 gene was inserted into vector pINA1312 for expressing and purification in the yeast *Y. lipolytica* system. *Y. lipolytica* is a widely used heterologous host in the food industry due to its unique physiological characteristics and enzyme system [28]. The recombinant strain Y7 successfully secreted Alyw202 with enzyme activity of 102.4 U/mL in GPPB medium (data not shown). The molecular weight of the recombinant Alyw202 was 38.3 kDa, same as that of the original enzyme (data not shown). This activity was nearly two times the activity obtained from the recombinant *Y. lipolytica* strain carrying ALYW201 gene. The obtained different activities of the two genes were due to the different specific activities. ALYW202 gene was a more suitable choice to achieve high alginate lyase activity.

### 2.4. pH Properties of Alyw202

**Figure 4:**
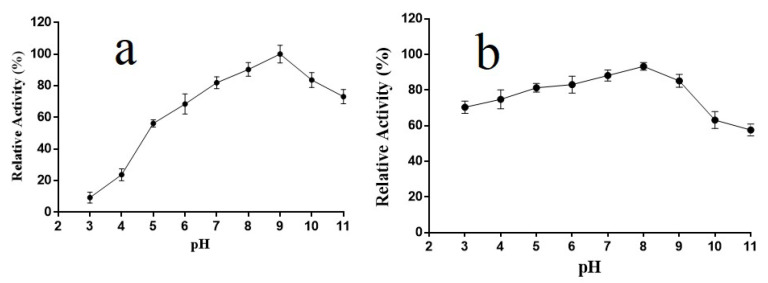
Effect of different pH on the relative activity of Alyw202. (**a**) Optimal pH of Alyw202; (**b**) Effect of different pH on stability of Alyw202. Data are given as mean ± standard deviation, *n* = 3.

The effect of pH on the activity of Alyw202 is shown in Figure 4. AlyW202 exhibited maximum enzyme activity at pH 9.0 (Figure 4a). Surprisingly, the endogenous AlyW202 maintained more than 80% activity in the solution over a broad pH range of 5.0–9.0, and it was still above 60% from pH 3.0 to 10.0 after 12 h incubation (Figure 4b). As previously reported, the alginate lyases from the PL7 family were active at neutral pH and show high activity in a narrow pH range [29,30]. Moreover, most of them exhibit instability under alkaline conditions. For instance, the AlySJ-02 and AlyIH only maintained their stability between pH 7.0–9.0 and 7.0–8.0 [31,32]. Zhu et al. characterized a pH-stable and mesophilic extracellular alginate lyase AlySJ-04 in a broad pH range of 4.0–10.0 [33]. Even compared with that of AlyW201 [13], a novel cold-adapted alginate lyase from the same *Vibrio* sp. W2, the pH-stable range of Alyw202 was also much broader. As shown in Table 2, Alyw202 possessed both a broader pH-stable range and higher specific activity. Thus, the results suggested that Alyw202 is a basophilic alginate lyase with excellent pH-stable properties suitable for industry application.

### 2.5. Temperature Properties of Alyw202

**Figure 5:**
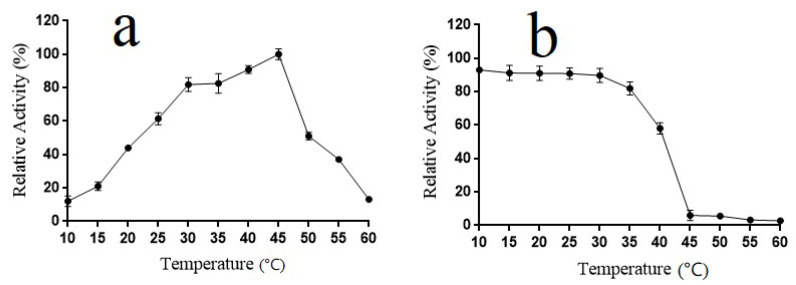
Effect of different temperatures on the relative activity of Alyw202. (**a**) Optimal temperature of Alyw202. (**b**) Effect of different temperatures on stability of Alyw202. Data are given as mean ± standard deviation, *n* = 3.

The intracellular enzyme Alyw202 showed maximum activity at 45 °C (Figure 5a) and was stable below 45 °C (Figure 5b). Thermostability analysis indicated that Alyw202 maintained more than 90% of the highest activity at a low temperature from 10 to 25 °C. Indeed, at temperatures below 40 °C, Alyw202 was remarkably stable; approximately 60% activity was maintained after incubation at 40 °C for 2 h, and then it was dramatically inactivated as temperature increased. The low-temperature activity seems to be the unique characteristic of alginate lyases shared by both intracellular Alyw202 and extracellular Alyw201 in *Vibrio* sp. W2. As previously reported, cold-adapted alginate lyases usually had lower thermostability below 35 °C, and above 50% of the highest activity at 20 °C [13,34,38,39]. Compared with other cold-adapted alginate lyases, Alyw202 showed higher percent of the maximal activity at 20 °C, meaning that Alyw202, a promising industrial cold-adapted enzyme, can efficiently degrade alginates in a way of energy conservation. In addition, it also can be inactivated selectively by increasing the temperature slightly during the catalytic process [35].

### 2.6. Effects of Ions on the Activity of Alyw202

**Figure 6:**
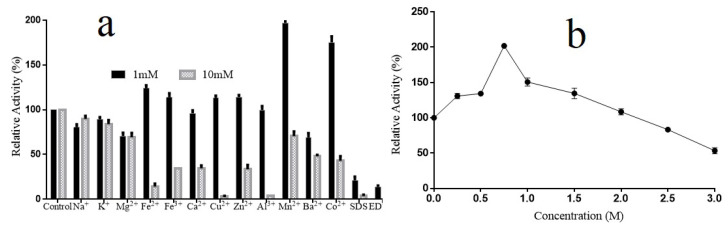
(**a**) Effects of metal ions, EDTA and SDS on the activity of Alyw202. (**b**) Effect of NaCl on the activity of Alyw202. Data are shown as mean ± SD (*n* = 3).

The effects of metal ions, EDTA and SDS on the activity of Alyw202 were determined at the concentrations of 1 and 10 mM separately (Figure 6a). At the concentration of 1 mM, Mn^2+^ and Co^2+^ significantly enhanced the activity by 196.8% or 175.1%, and Fe^2+^, Fe^3+^, Cu^2+^ and Zn^2+^ slightly increased the activity, while Na^+^, Mg^2+^ and Ba^2+^ showed slight inhibitory effects. However, with the presence of EDTA and SDS at the concentration of 1 mM the relative activity was significantly reduced. By contrast, at the concentration of 10 mM, almost all the metal ions, EDTA and SDS notably inhibited lyase activity, except Na^+^, K^+^, Mn^2+^ and Mg^2+^, which partially inhibited relative activity by 89.8%, 83.9%, 70.7% and 69.3%, respectively. Thus, the intracellular Alyw202 showed remarkable metal ion tolerance, and a moderate amount of Mn^2+^ can be applied to boost reaction at low temperature.

In addition, considering the significance of NaCl for enzymes originating from a marine environment, the activity of Alyw202 in the presence of NaCl at various concentrations was measured. As shown in Figure 6b, the activity of Alyw202 was significantly promoted by NaCl in a broad concentration range of 0–2000 mM and reached the maximum with 750 mM NaCl. Even at the NaCl concentration of 3000 mM the relative activity still maintained 50% of the highest activity. Therefore, the Alyw202 was a remarkable salt-tolerant and NaCl-independent alginate lyase like Alyw201 [38]. For most marine alginate lyases, NaCl of a certain concentration is crucial for the activation. For example, AlyA has no activity of alginate lyase in the absence of NaCl [11]. Aly08 was also dramatically enhanced by NaCl, and the activity reached about eight times higher than the activity in the absence of NaCl [15]. Therefore, this Alyw202 could be used directly in the degradation of brown algae from a marine environment without desalted preprocessing.

### 2.7. ESI-MS Analysis of Degradation Products

**Figure 7:**
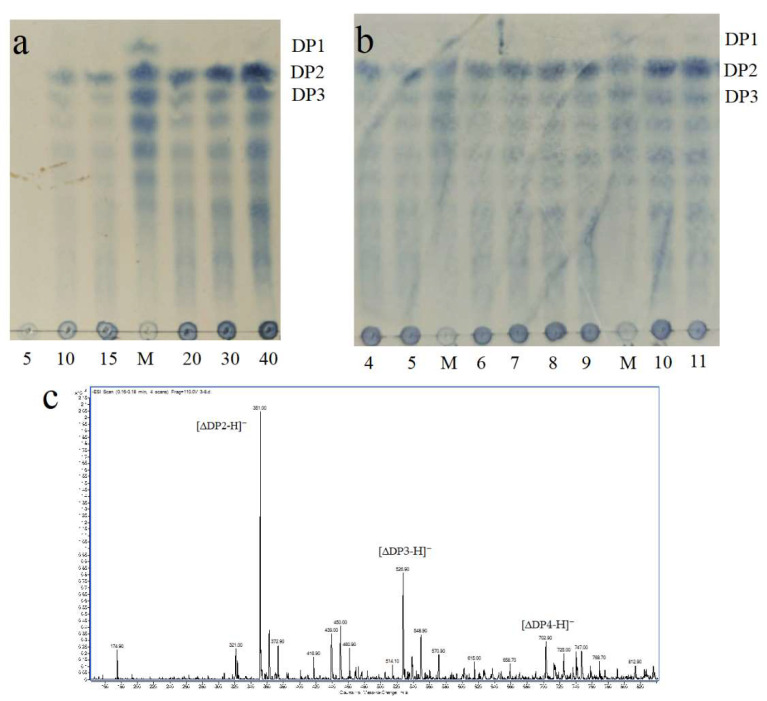
(**a**) Analysis of degradation products at different reaction times (min) by TLC. (**b**) Analysis of degradation products at different pH by TLC. (**c**) Analysis of degradation products by ESI-MS.

As shown in Figure 7a,b, the distributions of degree of polymerization were similar. Disaccharides and trisaccharides accounted for the biggest fraction. Among the degraded products at pH 4.0–11.0, disaccharides and trisaccharides were also the main components. The final degraded product at pH 7.0 was detected by negative-ion ESI-MS. As shown in Figure 7c, a series of oligosaccharides in peaks, such as disaccharides (351.00 m/z), trisaccharides (526.90 m/z) and tetrasccharides (702.90 m/z), were detected, among which disaccharides and trisaccharides accounted for the biggest fraction. Since there was no monosaccharide in the end products, the intracellular Alyw202 degraded alginate in an endolytic manner. This result was similar with other alginate lyases of the PL7 family, like the extracellular Alyw201, which also mainly produced oligosaccharides of DP2-DP5 in an endolytic manner [13,14,23,36]. Compared with other alginate lyases, Alyw202 showed high disaccharide-yielding levels.

As the minimum unit endowed with the peculiar antioxidant structure, disaccharides from alginate are thought to be the best antioxidants among AOS. However, disaccharides are generally present in low proportions (less than 50% of the total) in the products of most alginate lyases. Moreover, AOS with different degrees of polymerization (DPs) are very difficult to separate. Therefore, the characteristic of Alyw202 yielding high levels of disaccharides is of importance.

## 3. Materials and Methods

### 3.1. Materials, Strains and Mediums

Marine *Vibrio* sp. W2 was previously isolated from viscera of abalone. This strain was grown on sole-carbon source (ASC) solid/liquid medium. *Y. lipolytica* URA- transformants were screened on YNB plates. GPPB medium was used for recombinant enzyme production. The medium composition and culture conditions above were the same as mentioned before [40,41]. Sodium alginate (M:G = 1.66) derived from brown seaweed was purchased from Bright Moon Seaweed Group (Qingdao, China). PolyM and PolyG were purchased from Qingdao BZ Oligo Biotech Co., Ltd. The uracil mutant *Y. lipolytica* URA- strain and vector pINA1312 were kindly provided by Prof. Zhenming Chi, Ocean University of China.

### 3.2. Purification of Intracellular Alginate Lyase

The ASC culture was centrifuged at 5000× *g* for 10 min to collect strains and resuspended in 50 mM phosphate buffer (pH 7.0) before ultrasonic decomposition (250 W, 20 min) for intracellular activity assay. After centrifugation (12,000× *g*, 30 min), the intracellular alginate lyase was present in the supernatant. The intracellular alginate lyase was purified by using DEAE-Sepharose fast flow and Sephadex G-75 chromatography. The active fraction was analyzed by 12% (w/v) SDS-PAGE gel, and the protein concentrations were tested by using the BCA (Solarbio, Beijing, China) protein kit.

### 3.3. Enzyme Activity Assay

Alginate lyase activity assay was performed with 0.5% (w/v) polyM, polyG and alginate solution as substrate respectively as described in a previous study [41]. Enzyme activity was determined by increasing A235, as the hydrolysis reaction formed unsaturated double bonds. One unit (U) of enzyme activity was defined as the amount of enzyme required to increase A235 by 0.1 per minute, under the above conditions.

### 3.4. Bioinformatics Analysis of Alyw202

The genomic DNA of strain W2 was sequenced and annotated in Novogene Bioinformatics Technology Co. Ltd. (Tianjin, China). The sequence analysis showed a putative gene encoding alginate lyase without signal peptides with the ORF of 1053 bp. The ORF was analyzed by ORF Finder, and domain analysis was predicted using Conserved Domain Database (https://www.ncbi.nlm.nih.gov/). The online tool (https://web.expasy.org/compute_pi/) was applied to calculate the theoretical pI and Mw. Neighbor-joining phylogenetic tree was constructed based on the reported alginate lyases using MEGA version 7.0. Multiple sequence alignments among characterized PL7 family was obtained by using DNAMAN 6.0.

### 3.5. Secretory Expression and Purification of Alyw202

The ALYW202 gene with the XPR2 signal peptide gene was synthesized after codon optimization (Synbio Technologies, Suzhou, China). The synthesized alginate lyase gene was ligated into expression vector pINA1312 and then transformed into URA- strain [41]. The recombinant *Y. lipolytica* URA- harboring the pINA1312/ALYW202- XPR2 was cultured in a GPPB liquid medium for 84 h cultivation at 180 rpm and 30 °C. The recombinant strain Y7 was chosen for the highest extracellular activity. During the fermentation of Y7 in flasks, the alginate lyase activity and the biomass were detected every 12 h. All the data were collected in triplicate. The supernatant of strain Y7 containing the recombinant protein was loaded onto a Ni-NTA sepharose column (GE Healthcare, Chicago, IL, USA) equilibrated with lysis buffer (pH 7.0) as mentioned before. The binding buffer (50 mM phosphate buffer, pH 7.0, 500 mM NaCl, 20 mM imidazole, pH 7.0) was used to equilibrate the resin, while the elution buffer (50 mM phosphate buffer pH 7.0, 500 mM NaCl, 500 mM imidazole, pH 7.0) was used to obtain the intracellular protein. The active fraction was analyzed by 12% (w/v) SDS-PAGE gel.

### 3.6. Effects of pH and Temperature on Alyw202 Activity and Stability

The effects of pH on the recombinant enzyme activity were performed by incubating the purified enzyme in buffers (Na_2_HPO_4_-citric acid (pH 3.0–8.0), glycine-NaOH (pH 8.5–11.0)) under the assay conditions described before [13]. The pH stability depended on the residual activity after incubating at 4 °C in buffers with different pH (3.0–11.0) for 12 h. Meanwhile, the effects of temperature (10–60 °C) on the purified recombinant enzyme were measured at pH 8.0 to determine the optimal temperature. The thermal stability was determined by the remaining activity measured at 45 °C after incubating at temperatures ranging 10 to 60 °C for 12 h. All reactions were performed in triplicate.

### 3.7. Effects of Metal Ions, NaCl and Chemical Compounds on Alyw201 Activity

Mother liquors of metal ions, EDTA and SDS were prepared. The influences of metal ions on the activity of the recombinant enzyme were evaluated at 45 °C in the presence of various metal compounds with final concentrations of 1 and 10 mM. The Alyw202-catalyzing reaction without any metal ion was taken as control. Meanwhile, the catalyzing reactions of purified enzyme were also performed in alginate solution at 45 °C with different concentrations of NaCl (0–3000 mM). The maximum activity was taken as 100%. All reactions were performed in triplicate.

### 3.8. Analysis of Alyw202 Reaction Products

Alginate solution was hydrolyzed by Alyw202 for 40 min at pH 7.0 and 45 °C, and the degraded products were detected by TLC every 5 min. The degraded products at pH 4.0–11.0 were also detected. First, the products were analyzed with a solvent system (1-butanol/acetic acid/water 2:1:1, v/v/v) using a TLC plate (TLC silica gel 60 F254, Merck KGaA, Darmstadt, Germany). After spraying with sulfuric acid/ethanol reagent (1:4, v/v), the TLC plate was heated at 80 °C for 30 min to visualize the spots. To further determine the composition and degree of polymerization (DP), the degraded products pH 7.0 were desalted and investigated by ESI-MS after incubation at 45 °C for 40 min. The handling method was same as that of the previous study [13].

## 4. Conclusions

Few studies focusing on intracellular alginate lyases have been conducted. In this study, we have cloned, expressed and characterized a novel intracellular alkaline alginate lyase Alyw202, with a molecular weight of approximately 38.3 kDa. Alyw202 had the highest activity at 45 °C and more than 60% of the activity in a broad pH range of 3.0 to 10.0. Its high activity was a pH-stable, metal ion-resisted, NaCl-independent property. The ability to generate disaccharides as the main product makes it a good candidate for industrial use. This study will provide new insights for the development of novel biotechnologies for AOS production and separation. Further works will be focused on the molecular mechanism of the pH-stable property of Alyw202, along with the determination of its three-dimensional structure.

## Figures and Tables

**Table 1:** Summary of the purification of Alyw202.

Purification Step	Total Activity (U)	Total Protein (mg)	Specific Activity (U/mg)	Purification Fold	Yield (%)
Crude enzyme	6802.3 ± 0.5	86.4 ± 0.1	78.7 ± 0.6	1	100
DEAE-Fast Flow	5641.7 ± 0.6	12.3 ± 0.1	458.7 ± 0.4	5.8	82.9
Sephadex G-75	4611.3 ± 0.7	2.4 ± 0.1	1926.4 ± 0.4	24.5	67.8

**Table 2:** Comparison of Alyw202 with the related alginate lyases.

Name	Source	Molecular Weights (kDa)	Specific Activity	pH-Stable Range
Alyw202	This study	38.3	1926.4 U/mg	3.0–10.0
Alyw201	*Vibrio* sp. W2 [13]	38.0	876.4 U/mg	3.0–10.0
TsAly6A	*Thalassomonas* sp. [34]	83.9	15,960 U/μmol	6.6–8.95
TsAly7B	*Thalassomonas* sp. [35]	65	488.8 U/mg	7.3–8.6
ZH0-IV	*Sphingomonas* sp. [27]	113	12.3 U/mg	6.0–9.0
Algb	*Vibrio* sp. W13 [36]	55.05	457 U/mg	4.0–10.0
AlgNJ-04	*Vibrio* sp. NJU-04 [32]	50	2416 U/mg	4.0–10.0
rSAGL	*Flavobacterium* sp. [37]	33	4044 U/mg	-
AlgNJU-03	*Vibrio* sp. NJU-03 [25]	55.05	457 U/mg	6.0–9.0
